# Physiological Stress Reactions in Red Deer Induced by Hunting Activities

**DOI:** 10.3390/ani10061003

**Published:** 2020-06-08

**Authors:** Sofia Vilela, António Alves da Silva, Rupert Palme, Kathreen E. Ruckstuhl, José Paulo Sousa, Joana Alves

**Affiliations:** 1 Centre for Functional Ecology (CFE), Department of Life Sciences, University of Coimbra, Calçada Martim de Freitas, 3000-456 Coimbra, Portugal; vpsofia.a@gmail.com (S.V.); antonioalvesdasilva@gmail.com (A.A.d.S); jps@zoo.uc.pt (J.P.S.); 2 Unit of Physiology, Pathophysiology and Experimental Endocrinology, Department of Biomedical Sciences, University of Veterinary Medicine, Veterinärplatz 1, Vienna 2210, Austria; rupert.palme@vetmeduni.ac.at; 3 Department of Biological Sciences, University of Calgary, 2500 University Drive NW, Calgary, AB T2N 1N4, Canada; kruckstu@ucalgary.ca

**Keywords:** *Cervus elaphus*, plasma, feces, hair, glucocorticoids, hunting, stress

## Abstract

**Simple Summary:**

Game hunting is an activity largely practiced all over the world. Understanding its consequences on wildlife is crucial for the proper management and development of hunting directives. In this study, we examined stress levels in hunted wild red deer by assessing cortisol levels and its metabolites in multi-temporal biological samples. Overall, we found evidence for an influence on stress levels of red deer caused by repeated exposure to hunting events, which could have important implications on the sustainability and conservation of wild populations. Furthermore, our results highlight the use of hair samples as a useful long-term stress indicator.

**Abstract:**

Hunting activity is usually seen as a factor capable of causing an intense stress response in wildlife that may lead to short but also long-term stress. In the Lousã Mountain, Portugal, the population of red deer (*Cervus elaphus*) is the target of intensive seasonal hunting. We collected and measured cortisol (and its metabolites) in three tissues types (blood, feces and hair) from red deer hunted during two hunting seasons to evaluate the stress levels at different time windows. We also assessed the immunological and physical condition of the animals. We predicted that the hunting activity would act as a stressor inducing increased short and long-term stress levels in the population. Results showed an increase in hair cortisol levels during the months of harvesting. Surprisingly, the tendency for plasma cortisol levels was to decrease during the hunting season, which could be interpreted as habituation to hunting activity, or due to the hunting duration. Contrary to our predictions, fecal cortisol metabolites did not show any clear patterns across the months. Overall, our results suggest an influence of hunting activities on the physiological stress in red deer. In addition, hair seems to be useful to measure physiological stress, although more studies are required to fully understand its suitability as an indicator of long-term stress. Methodologically, our approach highlights the importance of simultaneously using different methods to assess short and long-term effects in studies on physiological stress reactions.

## 1. Introduction

Stress responses occur when an animal perceives an external noxious stimulus (stressor) such as predation, adverse weather, habitat change, or anthropogenic disturbances. In physiological terms, such stress reactions can be described as a cascade of endocrine secretions involving the hypothalamic-pituitary-adrenocortical axis (HPA) and sympatho-adrenomedullary system (SAS), wherein the adrenal glands play an important role [1,2]. These physiological mechanisms are induced to avoid, survive, or recover from an adverse condition [3], and involve the increase of glucocorticoid and/or catecholamine secretion leading to cumulative costs defined as allostatic load. When energy requirements of the organism exceed energy reserves, allostatic overload (type 1) happens [4]. Once the threshold is reached, the emergency response can be triggered, redirecting the animal towards survival and interrupting its normal life history. At a short-term scale, the rise of glucocorticoids (GC) induces physiological effects such as the regulation of the immune system, the increase in glucose created from the body’s energy stores, and contributes to memory consolidation [3,5,6]. However, long-term exposure to stressors may trigger type 2 allostatic overload, known as chronic stress [4]. At this point, there is sufficient or even excess energy consumption which can have negative consequences such as hypertension, inhibition of the immune system, promotion of neuronal cell death and reduction of body growth [3,5,6]. Additionally, with chronic, non-lethal stressors, animals can exhibit habituation to the stressor, which means that after a certain period of exposure the animals no longer perceive the stimulus as noxious. Therefore, at this point, the rise of GC is no longer triggered [2].

The quantification of glucocorticoids is an important tool to study the neuro-endocrine stress axis and can provide insights into an animal’s well-being as well as a better understanding of ecological and evolutionary processes, which can be useful for conservation purposes [7,8,9,10,11,12]. The determination of cortisol or corticosterone in plasma is widely used as an indicator of stress [1,12]. This measure represents the instant stress status of an animal and can be used as a tool for experimental studies to understand the effects of specific stressors such as predators or weather conditions [12,13,14]. However, since this method requires the handling of animals for blood collection, which in itself represents a stressful situation for animals, new methods with non-invasive sampling have been developed. Above all, analysis of glucocorticoid metabolites in feces has been established as a validated, successful alternative to blood, in several studies [1,9,10,15,16]. In red deer, using fecal samples, maximum levels of cortisol metabolites are usually recorded with a delay of approximately 18 h after the disturbance [17]. In addition, cortisol in hair has been used as an indicator of stressful conditions [18,19]. Some recent studies suggest that hair shows levels of GC that are accumulated for some weeks to months, depending on the species [20,21,22,23].

It has been shown that hunting is capable of causing an intense stress response in wildlife, which can significantly affect animal welfare. Bateson and Bradshaw [24] showed that hunting triggers physiological effects in red deer (*Cervus elaphus*), such as disruption of muscle tissue and alterations of concentrations of β-endorphin. Moreover, the same authors demonstrated that cortisol levels increased considerably when animals were exposed to this kind of harvesting. Although its effects are poorly explored, hunting activities, due to their seasonality, can result in repeated and prolonged stress. Recently, a study suggested that active hunting events using hounds or involving great densities of people can have a stronger impact with higher cortisol levels in cervids than calmer stalking [25].

In Portugal and Spain, the main hunting process used is a non-selective process called “montaria”, which involves the release of packs of dogs (usually 150 to 300 dogs), that will chase the animals during a maximum of five hours, and move them toward the hunters, positioned at the edge of the hunting area [26,27]. Red deer is an important game species in Portugal, and its exploitation is mostly done with this type of hunting process [26]. Due to the effects that this hunting practice can have on wildlife, its ethical and economical effects have been debated [28]. Notwithstanding the revenue that hunting can generate, this activity can also lead to several consequences in terms of disruption of age and social structure, animal dispersion and life expectancy [29,30]. Moreover, and as suggested in some studies [31,32], in wild populations, hunting may introduce an element of artificial selection. Thus, hunting activities can induce significant changes in demography and population genetics, such as social disruption or consequences on the gene pool of hunted populations [32,33]. The sustainability and conservation implications of this practice, which include effects on demography, genetic pool, behavior and general health, highlight the importance of management and regulation of hunting activities [5,27,33].

The main aim of this study was to investigate the impact of hunting activities on a wild population of red deer using measurements of glucocorticoids in plasma and hair and its metabolites in feces, all collected from hunted animals. We thus indirectly evaluated the stress levels of each hunted animal from a few months before the hunting event until its death. Given the wide effect that a stress response can have on the body, the physical and immunological condition of the animals was also evaluated.

## 2. Materials and Methods

### 2.1. Study Area and Red Deer Population

The study took place at Lousã Mountain (40°3′ N, 8°15′ W) and surrounding hunting areas, located in Central Portugal. The climate in this Mediterranean area is characterized by hot and dry summers and rainy winters [34]. With an altitude that ranges from 100 to 1205 m, this mountainous region is predominantly composed of plantations of coniferous and broadleaf trees, and large shrubland areas [26].

The red deer population in this region is the result of a reintroduction program that occurred between 1995 and 1999 with the release of 96 animals. Currently, this species occupies around 435 km² of the Lousã Mountain and surrounding areas, with an estimated density of 5.6 deer/km² during the rut season [35,36]. The calving season of this population occurs in May/June and rutting in mid-September to the end of October. With females being 37 ± 3% (mean ± SE) smaller than males, this species shows a very marked sexual dimorphism in body size. In addition, the sexual segregation outside the breeding season results in a matriarchal society wherein the females adopt a more philopatric behavior and males tend to disperse [35,37]. Since there are no natural predators, red deer are mainly preyed upon by feral dogs. This species is one of the most hunted big game species of the Lousã Mountain region, which includes 12 hunting areas, with red deer hunted in seven of them since the start of hunting in the region in 2006/2007 [26].

### 2.2. Data Collection

The study was performed during two hunting seasons (2013/2014 and 2014/2015), enabling the collection of samples from 80 red deer (38 adults: 26 females and 12 males; 30 sub-adults: 14 females and 16 males; and 12 young). The samples were collected during autumn (October and November) and winter (January and February) from red deer hunted in six “montarias” in three contiguous hunting areas: ZCM (Municipal Hunting Area) of Lousã (three hunting events—34 animals), ZCM of Vila Nova (one hunting event—7 animals) and ZCA (Associative Hunting Area) of Miranda do Corvo (two hunting events—39 animals) located in the Lousã Mountain area. No hunting events were conducted during the month of December. Post-mortem examination was made in situ, one to four hours after the animals were killed, and included the collection of blood, feces directly from the rectum, hair from the dorsal region and the metatarsus, and the recording of the sex and age class (based on animal size, body conformation and characteristics of antlers) of each animal. In addition, in November, fecal samples (*n* = 11) were also collected non-invasively during behavioral observations in the non-hunting area (i.e., area where hunting is not allowed) located in the central part of the Lousã Mountain, to use as the control.

Blood samples were taken directly from the heart into EDTA-tubes and centrifuged at 2000× *g* for 5 min. Plasma was collected and frozen at −20 °C in multiple aliquots. Feces, hair, and the metatarsus were frozen and stored at −20 °C for subsequent analyses.

### 2.3. Steroid Extraction and Quantification

Five ml of diethyl ether was added to each plasma sample (0.5 mL). After being shaken and centrifuged (2500× *g*, 15 min), the samples were frozen. Afterwards, the liquid component was transferred to a glass vial and dried under a stream of nitrogen (40 °C). This procedure was performed twice to increase the recovery of the extraction process. The combined and dried down extracts of each sample were dissolved in 0.5 mL of assay buffer and an aliquot analyzed by a cortisol enzyme immunoassay (EIA), as described in detail before [38]).

From each dried fecal sample 0.2 g were taken and mixed with 4 mL of methanol (100%) and 1 mL of water. The samples were shaken for 30 min and centrifuged at 2500× *g* for 15 min [39]. A group (with a 3α-11-one structure) of fecal cortisol metabolites (FCMs) was measured using an 11-oxoetiocholanonolone EIA. The utilized biotinylated label and the antibody (including its cross-reactivity) have been previously described in detail [40]. The assay has been successfully validated for use in red deer [41].

Hair samples were cut into fragments (<0.5 cm), washed with 5 mL 100% n-hexane to remove any lipids and potential external contamination, and air dried. Hair samples (0.05 g) were mixed with 5 mL of 100% methanol and incubated for 72 h for glucocorticoid extraction. After transferring the supernatant into a new glass vial, the organic solvent was evaporated at 40 °C using a stream of nitrogen. The extracts were dissolved in 0.5 mL of assay buffer and analyzed with a cortisol EIA [38,42].

### 2.4. Physical and Immunological Conditions

The physical condition of animals was assessed using bone marrow fat (BMF). The BMF was determined using the metatarsus, from where the bone marrow was extracted and weighed (± 0.0001 g). The bone marrow samples were then oven-dried at 60 °C and reweighed. BMF was determined as BMF = (the weight of oven-dried marrow/fresh marrow) × 100 [43].

To evaluate the immunological state of an individual, we used the blood collected in the field to make blood smears in the lab, followed by staining [44]. White blood cell counts and identification (100 cells per smears) were performed using a Nikon Eclipse Ni microscope (Nikon Instruments Europe B.V., Amsterdam, Netherlands). The examination and classification of blood cells were made based on morphological criteria and properties of staining, allowing the identification of five types of white blood cells, namely lymphocytes, neutrophils, eosinophils, monocytes and basophils, respectively [45].

### 2.5. Statistical Analysis

The correlation between the concentrations of GC in the different sampling tissues (i.e., plasma, feces and hair) was tested using Pearson’s correlation. To analyse the physiological stress reactions, general linear models (LM) were used to test the effects of sex, age-class and month (independent variables) on the different GC levels (cortisol in plasma and hair, FCMs; dependent variables). Additionally, to evaluate the influence of the physical condition and the immunological status of the animals on GC levels, BMF and percentage of lymphocytes (the most abundant white blood cells (WBC)) were included in this analysis as independent variables. The concentrations of GC or its metabolites were transformed using a log transformation. BMF and WBC were logit transformed to achieve an approximation of a normal distribution and to reduce heterogeneity [46]. Since no significant interaction effects between the independent variables were found, only the main effects were used in the final models. The year had no significant effect on the concentrations of cortisol in plasma (F_(1,48)_ = 1.989; *p* = 0.165) or hair (F_(1,74)_ = 0.022; *p* = 0.882), nor on the concentrations of FCMs (F_(1,72)_ = 2.691; *p* = 0.105). Therefore, data of both years were pooled.

To evaluate the physical and immunological conditions of red deer, we tested the effects of sex, age-class and month (independent variables) on BMF and WBC (dependent variables) using general linear models (LM).

The results are expressed as mean ± SE and 95% confidence intervals (CI). All the statistical tests were considered significant when *p* < 0.05. The statistical analyses were performed using IBM.SPSS^®^, version 22 (IBM Corporation, New York, NY, USA).

## 3. Results

### 3.1. Physiological Stress Reactions

Concentrations of plasma and hair cortisol and its metabolites in feces were not correlated (Table 1). There was only a weak, but not significant correlation of cortisol in hair with fecal cortisol metabolites (FCMs).

**Figure 1:**
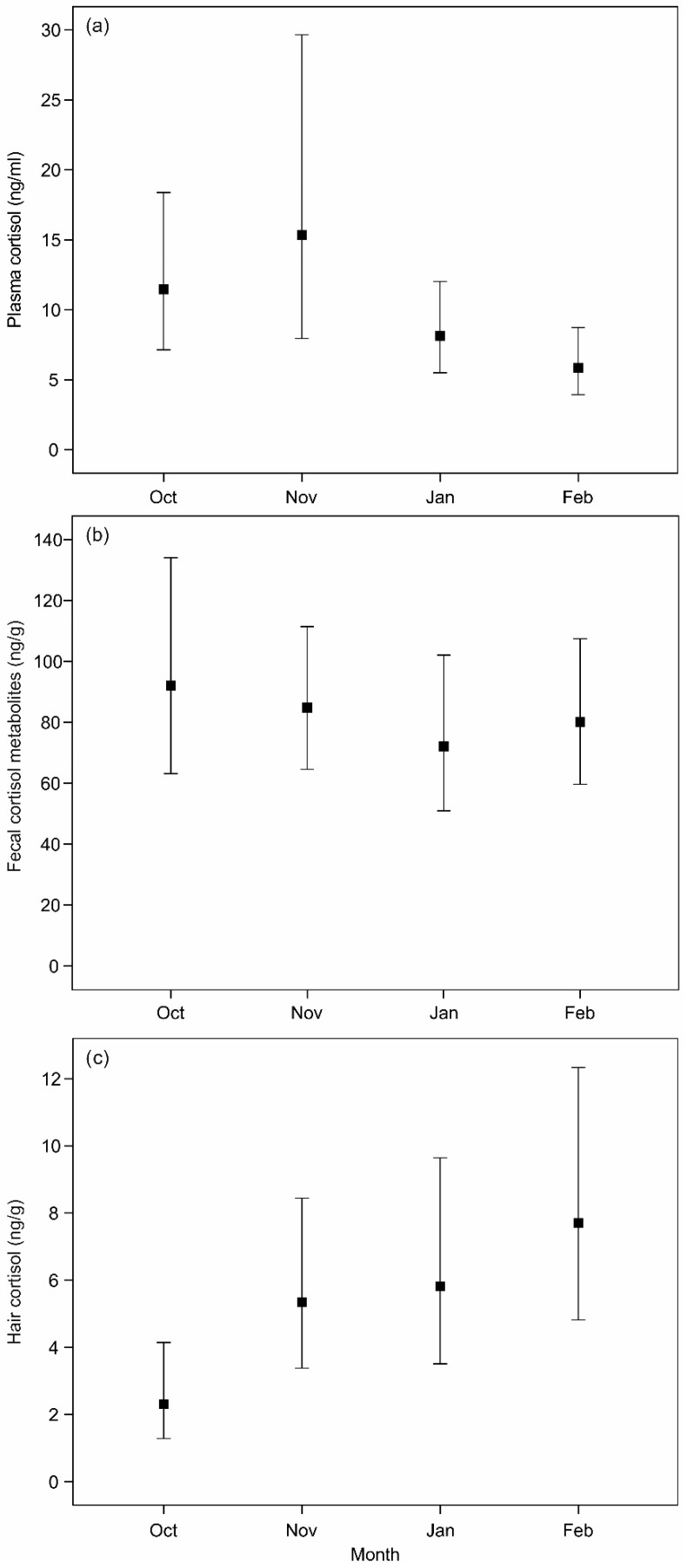
Patterns of cortisol (metabolite) levels in (**a**) plasma (**b**) feces and (**c**) hair during the hunting season (October to February). The values represent the estimated means from the general linear models and the bars represent the 95% confidence intervals.

Concentrations of plasma cortisol did not show differences between sex (F_(1,43)_ = 0.361; *p* = 0.551) or age classes (F_(2,43)_ = 0.074; *p* = 0.929). Comparison of plasma cortisol concentrations between months showed marginally significant differences (F_(3,43)_ = 2.809; *p* = 0.051) between the first and last months of the hunting season (Figure 1a). These results were not affected by BMF levels (F_(1,42)_ = 0.029; *p* = 0.866). However, the percentage of lymphocytes was associated with the concentration of plasma cortisol (F_(1,42)_ = 5.947; *p* = 0.019; β = 2.553). Once we controlled for the percentage of lymphocytes, the differences between months became more pronounced (F_(3,42)_ = 4.356; *p* = 0.009). FCM levels did not differ significantly in terms of sex (F_(1,67)_ = 0.769; *p* = 0.384), age class (F_(2,67)_ = 1.589; *p* = 0.212) or month (F_(3,67)_ = 0.367; *p* = 0.777; Figure 1b). Moreover, FCM levels were neither significantly influenced by the BMF levels (F_(1,65)_ = 3.206; *p* = 0.078) nor by the percentage of lymphocytes (F_(1,44)_ = 1.151; *p* = 0.289). Additionally, FCM values from hunted animals were higher (99.72 ± 11.33 ng/g) than those found in wild animals at control areas (51.17 ± 6.89 ng/g), for the same month (i.e., November). Regarding hair samples, levels of cortisol did not differ between sex (F_(1,69)_ = 2.959; *p* = 0.090) and age classes (F_(2,69)_ = 1.570; *p* = 0.215). Cortisol levels in hair differed between months (F_(3,69)_ = 3.805; *p* = 0.014), with the highest values recorded in February and the lowest in October (Figure 1c). Cortisol levels in hair were neither influenced by BMF (F_(1,68)_ = 1.659; *p* = 0.202) nor the percentage of lymphocytes (F_(1,46)_ = 2.371; *p* = 0.130), and the differences and patterns between months remained the same even after controlling for BMF levels and the percentage of lymphocytes.

### 3.2. Physical and Immunological Conditions

**Figure 2:**
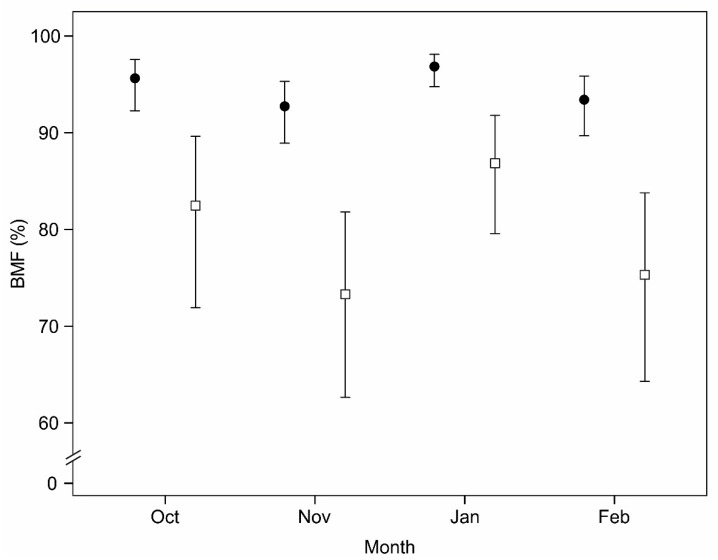
Patterns of bone marrow fat (BMF) levels for red deer females (black circles) and males (white squares) during the different months of the hunting season. The values represent the estimated means from the general linear models and the bars represent the 95% confidence intervals.

In terms of physical condition, measured by the BMF index, we found significant differences between the sexes (F_(1,72)_ = 43.370; *p* < 0.001), age classes (F_(2,72)_ = 6.842; *p* = 0.002) and months (F_(3.72)_ = 3.288; *p* = 0.025). Females had higher BMF values than males in all months (Figure 2). The differences obtained between age classes were mainly due to the poorer physical condition of calves (79.6; 95% CI [68.2, 87.6]) when compared with sub-adults (92.9; 95% CI [90.0, 94.9]) and adults (92.8; 95% CI [90.0, 94.8]). Regarding months, the animals had higher values of BMF in January (93.4; 95% CI [89.9, 95.8]) than in November (85.5; 95% CI [79.6, 90.0]). Lastly, white blood cells did not differ between sexes, age classes or across months (*p* > 0.05 for all models).

## 4. Discussion

Our results confirmed that concentrations of cortisol (or its metabolites) in plasma, feces and hair samples taken at the same time are not correlated. These results were expected since each sample matrix provides information about the endocrine state at different times. GC levels in plasma reflect an immediate physiological state [12], while feces provide information about the endocrine state a certain time before the sample collection [11] and hair is supposed to reflect the status of an accumulated period of some weeks to a few months [22]. Therefore, each biological sample matrix reflects distinct time-windows and thus complementary information can be gained.

The levels of cortisol and its metabolites did not differ between sex or age classes. These results were in agreement with those obtained by Huber et al. [17] who did not find differences in the concentration of FCMs between female and male red deer. However, other studies described sex-specific differences in glucocorticoid levels for some ruminant species [47,48] as well as age variations [49]. Regarding the influence of the reproductive state on stress levels, changes in cortisol levels were reported in red deer, with older females having higher cortisol levels in late gestation than non-pregnant females [50]. However, for reindeer (*Rangifer tarandus*) no differences were found in plasma cortisol concentrations between adult males, barren, and pregnant females [51]. Although the reproductive state of the females was not assessed in the present study, previous long-term data from the same study area has shown that during the sampling period more than 80% of females were usually pregnant (unpublished data), which means that probably more than 80% of the sampled females for this study were also pregnant. This fact, together with the absence of differences between males and females or age classes suggests that all animals were exposed to the same levels of stress during the sampling period. In fact, considering the type of hunting process used in our study area, which is not targeting any particular sex or age class, the obtained results are in agreement with our predictions. In fact, the absence of differences between males and females or age classes suggests that all animals were exposed to the same levels of stress during the sampling period.

We found a trend in plasma cortisol concentrations to decrease during the hunting season from November to February. A decrease of GC concentrations after regular and frequent occurrences of a stressor is often an indication of acclimation [2], as observed in some studies with Brahman cattle (*Bos taurus indicus*) and Magellanic penguins (*Spheniscus magellanicus*) [52,53]. However, besides the fact that we are dealing with a major stressor in our study, we need to consider the possible influence that this specific hunting method may have on our results. Since we have no information for how long deer were chased by the dogs before being killed, the cortisol values in plasma could reflect different chasing periods. Furthermore, the observed trend for plasma may also be influenced by other factors, such as food intake or/and any environmental disturbance [54,55,56], which make an interpretation more difficult. Although an influence of circadian rhythm in cortisol levels has been reported [55], the hunting events in the study area occurred within the same period of the day, decreasing the possible effect of daily circadian rhythm in our results.

Besides the trend obtained for plasma cortisol concentrations, the results also showed a positive correlation of plasma cortisol levels and the percentage of lymphocytes. We expected the opposite, namely a decrease of the percentage of lymphocytes with the increase of the stress levels, mainly due to the effect of stress as an immune suppressor [57]. Instead, our results were more in line with the immune-enhancing character of acute stress, promoting the passage of leukocytes from the blood to other parts of the body, while chronic stress induces immune suppression [57,58,59]. Although this interpretation requires caution given the existence of some unevaluated health-related factors, it points to the important relation between white blood cells and cortisol levels, and the need for more studies approaching this interaction.

We did not find differences in FCM concentrations across the sampled months. However, a seasonal pattern of GC levels in cervids is suggested by some authors who documented higher values in colder months than in warmer months of the year [17,60]. The minor influence of Mediterranean mild winters [34] where the occurrence of snow is uncommon and food availability is not significantly affected may have contributed to the lack of a seasonal pattern in our FCM levels during the hunting season. In fact, some studies in Mediterranean red deer [61] and roe deer (*Capreolus capreolus*) [62] suggested summer as the season with the most energetic constraints due to decreased food quality and quantity due to hydric stress. These results point, although weakly, given the small control sample size in terms of control group, to an influence of hunting in the stress levels. These results are in agreement with those reporting higher FCM levels in chamois (*Rupicapra rupicapra tatrica*) in areas with high disturbance than at low disturbance locations [16]. Hair cortisol concentrations differed significantly across months, with an increase from the beginning (October) to the end (February) of the hunting season. Cortisol levels recorded in February were significantly higher than the ones obtained in October. Taking into account that the molt from the summer to the winter coat is gradual, especially in adults where the development of new hair can occur before shedding the old one [63], our samples from October included new hair, which began to grow in September and October, and hair from the summer months which had not been shed yet. Furthermore, once hair follicle activity is reduced in February, because the end of the winter season is getting close and the winter coat is fully grown [63], cortisol measured in hair that was sampled in this month should largely reflect the conditions from the previous three months [64]. Therefore, the increase in cortisol levels across months may be an indication of a period of prolonged stressful conditions induced by hunting activity, which is supported by the higher FCM levels in individuals from impact areas than from control sites found in our results. Similarly, Caslini et al. [65] found that hair cortisol levels in the same species (*Cervus elaphus*) were higher in greater density areas associated with more difficult environmental conditions and higher levels of anthropogenic disturbance (such as tourism). Our results are in agreement with those reported in the mentioned study which suggested that long-term HPA axis activity and allostatic load, as a consequence of higher densities, anthropogenic disturbances and/or environmental conditions in red deer populations, can be evaluated using cortisol hair levels as an index [65]. In addition, Bryan et al. [66] also documented higher hair cortisol levels in heavily hunted wolves (*Canis lupus*) than in wolves with lower hunting pressure. Hair cortisol seems to be a good indicator of long-term stress and has gained importance as a novel method to assess stressful conditions [19,22]. Hair can often be collected without capture and handling of the animals (i.e., hair traps). Collection of hair for hormone analyses may thus be a useful, non-invasive tool to monitor prolonged stressful conditions. Moreover, the fact that cortisol levels in hair may provide a long-term endocrine profile [67,68] can be extremely useful to study chronic stress and animal welfare [69]. On the other hand, taking into account our results regarding plasma cortisol levels and FCM concentrations, hair might not be suitable at capturing short-term stress levels.

Based on our results, and previous work, increased hair cortisol concentrations in our red deer population seem to be a consequence of hunting activities. However, considering that our study was focused on a wild population, there are other factors that may be contributing to the GC levels obtained in hair, like temperature, food availability or season [17,60,70]. As the energetic balance is a crucial factor in the ability of the animal to respond effectively to certain stressors [4], the body condition, a measure of the long-term energetic reserves [26,71], can have an influence on cortisol levels. Therefore, considering this bidirectional interaction, not only can stress affect body condition, energetic parameters could also be important in dealing with stress. Our results did not show any correlation between cortisol levels and BMF, which is a measure of physical condition, making the influence of season and/or food availability on cortisol levels unlikely. Moreover, the source of cortisol accumulated in hair is unclear, and some possible explanations have emerged. Keckeis et al. [72] reported a local production of GC in the hair follicles of guinea pigs, however, how this mechanism is modulated is still unknown. Recently, experimental evidence was provided in domestic sheep (*Ovis aries*) that mechanical irritation of the skin significantly increased hair cortisol concentrations [73]. Another study suggested the existence of a cutaneous HPA axis, able to synthesize and secrete cortisol, as well as negative feedback regulation by cortisol under corticotropin-releasing hormone (CRH) expression [74]. The uncertainty about the origin of cortisol in hair leads to an additional caution in the interpretation and analysis of GC levels in these types of samples [75,76]. To decrease the influence of confounding factors in our study, hair samples were taken from the dorsal region in all the individuals. However, further investigation would be very important to clarify whether cortisol concentrations are affected by the level of hair pigmentation as well as body area, hair type, or if there is any pattern along the hair shaft as suggested by some studies [18,20,21]. In addition, it is also relevant to emphasize the importance of the combined use of different indicators of stress to obtain more complete and precise information about the stress conditions of wild populations [10]. Plasma, feces and hair are complementary tools, which can provide information from different time-windows, allowing a better evaluation of the effects of human activities, like hunting, on the physiological stress response.

In terms of physical condition, our results showed that females were in better physical condition than males. This could be due to differential costs of reproduction for each sex, with males going through a phase of hypophagia and high activity levels during the rut [26,37]. Young individuals also had lower BMW indices than adults, which may be the result of a greater investment of these animals into growth [77]. Despite the observed differences in physical condition, and contrary to our predictions, the stress parameters we measured were not associated with physical condition. However, Cabezas et al. [78] reported lower values of body condition in animals with high GC levels in wild rabbits (*Oryctolagus cuniculus*). The absence of an association between stress levels and physical condition may indicate that the studied red deer population had enough fat reserves to cope with the stress induced by hunting activities.

## 5. Conclusions

The ability of plasma, feces and hair to provide multi-temporal information about physiological state proved to be very useful in the present study. Although the use of different biological samples increased the difficulty in the interpretation of our results, it allowed a broader panorama, which was more complete and reliable to understand given such a complex topic like stress reactions. We found evidence that repeated exposure of our red deer population to game hunting activity had an impact on stress levels, which can have important consequences for sustainability and conservation of this species. Specifically, stress can affect population dynamics, by changing foraging and breeding behavior, animal welfare, and, ultimately, the evolutionary processes, by changing individual fitness and selection [2,5,6]. Thus, exploring these topics is crucial to understanding the implications of hunting for the conservation of this species and to improve hunting management activities. Our study highlights the fundamental and broad role of stress in wildlife, emphasizing the need for more studies capable of clarifying how different biological matrices may be useful to evaluate the impacts of human pressure on wildlife, both in terms of stress level and stress processes.

## Figures and Tables

**Table 1:** Pearson correlations between the concentrations of cortisol (metabolites) in plasma, feces and hair.

	Feces (*n* = 74)	Hair (*n* = 76)
Plasma (*n* = 50)	r = −0.152	r = −0.085
	*p* = 0.307	*p* = 0.561
Feces (*n* = 74)	-	r = −0.217
		*p* = 0.071
